# Use of Local Perforator Flaps for Post Burn Reconstruction

**Published:** 2012-01

**Authors:** Nikhil Panse, Parag Sahasrabudhe, Yogesh Bhatt

**Affiliations:** 1 Department of Plastic Surgery, BJ Medical College and Sassoon Hospital, Pune, India; 2 Department of Plastic Surgery, Medical College and SSG Hospital, Baroda, India.

**Keywords:** Perforator flaps, Propellar flaps, Burn reconstruction

## Abstract

**BACKGROUND:**

Mainstay of post-burn reconstruction is release and split skin grafting. Pedicle flaps are cumbersome to the patient, require multiple procedures and hospitalization. Free flaps are technically demanding and facilities are not universally available. Here we evaluated the local perforator flaps for post-burn reconstruction.

**METHODS:**

We have used sixteen perforator propellar flaps for post-burn reconstruction for various areas of body.

**RESULTS:**

All flaps did well without any recontracture and need of splintage.

**CONCLUSION:**

Local perforator flaps should be considered as one of the primary treatment options for post burn reconstruction.

## INTRODUCTION

Incisional and excisional release and skin grafting are the mainstays of post burn reconstruction. However using skin grafts in burn reconstruction has its own sets of problems. They are prone to contraction and recurrences that are common after contracture release and grafting. So there is a prolonged need of splintage and physiotherapy in the post-operative period.^1^

Flaps are better for resurfacing the defects after post-burn contractures. Flaps do not need rigorous post-operative physiotherapy or splintage and grow with age especially in children. However, local unscarred tissue is not available most of the times.^1^ Harvesting a tissue from the burnt area is possible, but there are increased chances of flap failure. Inclusion of a perforator in the flap of harvesting a perforator flap improves the vascularity of the flap, the arc of rotation and reduces the dog ear formation. ^1^ It has also the advantage of increased positional comfort as compared to distant flaps like the abdominal or the groin flaps. Harvesting a perforator flap is technically simple and gives good results. It should be considered as a primary option in treatment of post-burn contractures and sequel whenever possible. In this study, we evaluated the local perforator flaps for post-burn reconstruction.

## MATERIALS AND METHODS

We operated 16 cases of post-burn sequel including burn contractures, post burn raw areas, marjolins ulcers and post electric burn defects over multiple areas. The flaps were done for different areas of the body including knee, thigh, axilla, elbow, wrist and fingers. All the patients were managed using local perforator flaps (Table 1).

**Table 1:** Cases of post-burn sequel including burn contractures, post burn raw areas, marjolins ulcers and post electric burn defects over multiple areas.

**Sr.** **No**	**Sex**	**Age** **(Yrs)**	**Area affected**	**Type of burn**	**Duration since burn**	**Type of perforator flap**	**Source vessel**	**Number of perforators**
1.	M	34	Tendoachillis defect	Electric burn	3 months	VY advancement flap	Peroneal & Posterior tibial	2
2.	F	18	P.B.C Axilla	Flame burns	4years	Islanded Perforator flap	Circumflex scapular	1
3.	M	22	P.B.C Wrist	Electric burns	5 months	propeller flap	Radial artery	1
4.	M	28	Dorsoulnar wrist defect	Electric burns	3 weeks	Perforator plus flap	Posterior interosseus artery	1
5	M	43	P.B.C Knee	Flame burns	6 years	Islanded Perforator flap	Genicular artery	1
6.	F	20	P.B.C Elbow	Flame burns	1.5 years	Multilobed propeller flap	Radial artery	1 with subcutaneous pedicle
7.	F	28	P.B.C Axilla	Flame burns	7 years	Islanded Perforator flap	Circumflex scapular	1
8.	F	35	P.B.C Elbow	Flame burns	3years	Multilobed propeller flap	Radial artery	1 with subcutaneous pedicle
9.	F	38	P.B.C Knee	Flame burns	2years	Islanded Perforator flap	Genicular artery	2
10.	F	42	p.B.C Knee	Flame burns	3years	Islanded Perforator flap	Genicular artery	1
11.	F	45	Post burn Marjolins ulcer	Flame burns	13years	VY advancement flap	Descending branch of lateral circumflex femoral artery	1
12.	F	32	Little finger contracture	Flame burns	2 years	Propellar flap	Digital Ulnar artery	1
13.	M	11	Little finger contracture	Flame burns	3years	Propellar flap	Digital Ulnar artery	1
14.	M	45	Little finger contracture	Flame burns	3 years	Propellar flap	Digital ulnar artery	1
15.	M	36	P.B.C Wrist	Electric burn	8 months	Propellar flap	Ulnar artery	1
16.	F	35	P.B.C Knee	Flame burns	5 years	Islanded Perforator flap	Genicular artery	1

The operative steps for harvesting of a perforator flap were as follows: We did a preoperative perforator identification by hand held Doppler with an 8 Hz probe in all the patients. We considered it desirable though not mandatory to identify the perforator preoperatively. Contracture release/debridement and flap elevation was done under tourniquet control. The width of the flap was dependent on the width of the defect and the angle of rotation of the flap. The width of the flap was equal to the vertical height of the defect if the flap rotated 90 degrees; while it was equal to the antero-posterior diameter of the defect in case of 180 degrees rotation.^2^ The superior margin of the flap was determined intra-operatively, after mobilization of the pivot perforator. The length of the flap proximal to the pivot perforator was equal to the length from the edge of the defect to be closed plus one cm. This one cm was added to allow tension free closure.

After creation of defect, the incision was made through skin and deep fascia. Because dominant or primary cutaneous arteries emerged from the deep fascia near where the fascia was fixed to bone or anchored by inter-muscular septa, these sites, as revealed by natural skin crease lines, were explored first. Sub-fascial sharp dissection proceeded until the perforator was reached. Sharp dissection preserved the sub-fascial plexus.^2^This plexus was less important than the supra-fascial one, but we feel that it had a role in the blood supply of the deep fascia. The perforators were dissected free of surrounding structures. The pivot perforator was completely mobilized and its integrity was determined. The anterior incision was opened and the proximal end of the flap was measured and cut. At this stage, the flap attached the body by the perforators. All the extra perforators were clamped with micro clamps. The viability of the flap, shown by refilling of vessels and bleeding from the flap, was observed. If viability was judged good, the clamped perforators were ligated and cut.

**Fig. 1:**
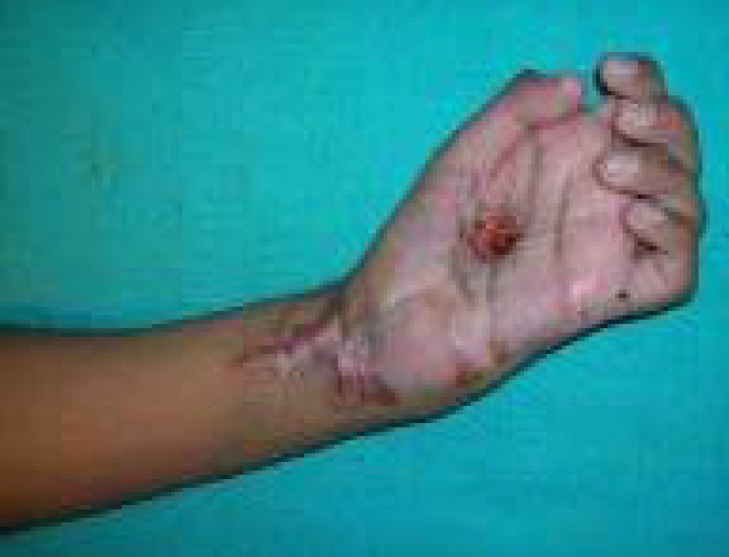
Post electric burn wrist contracture

**Fig. 2:**
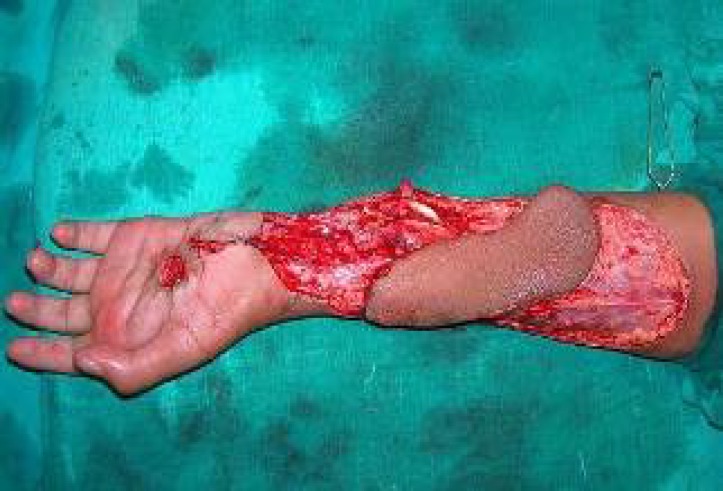
Harvested radial artery perforator flap.

**Fig. 3:**
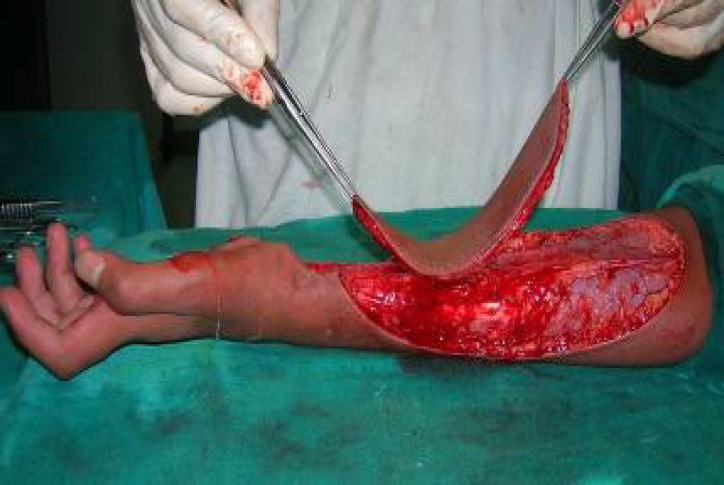
Flap islanded on perforator of radial artery

**Fig. 4:**
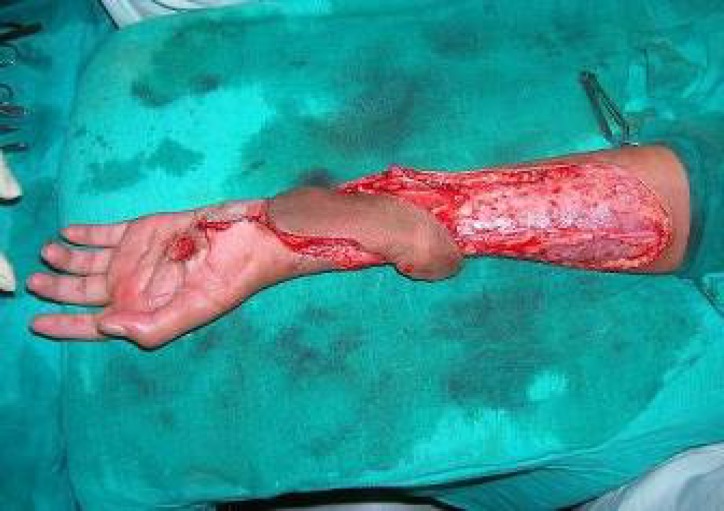
Flap rotated into the defect.

**Fig. 5:**
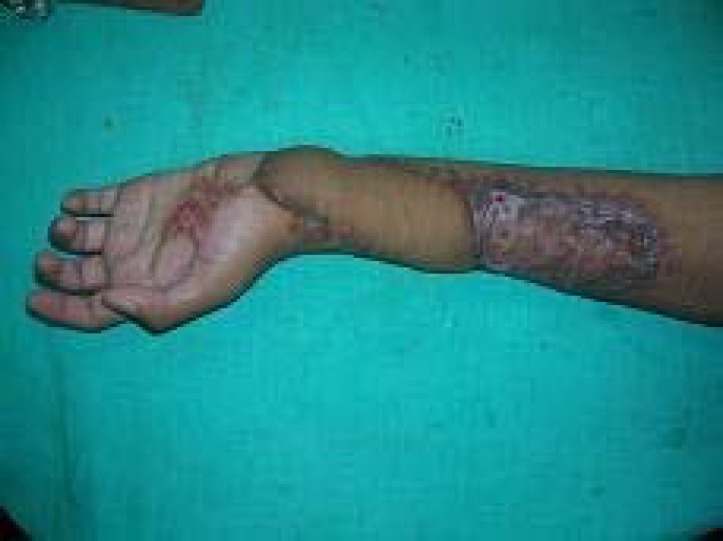
Well settled radial artery perforator flap.

A reliable perforator was believed to have the ability to expand its perfusion over its territory after perforator flap elevation. Based on the clinical experience, the reliable was a perforator that sprouted from carrier muscles or septum with a visible pulsation. It had the peculiar ability to overcome the angiosome barrier through sub-dermal network. Now, the flap was an island attached only by its pivot perforator-artery and its venae comitantes. The flap was rotated on the axis of this perforator up to 180 degrees to cover the defect or was advanced into the defect. After rotation or advancement, the viability of the flap was reassessed. If there was any compromise, there might be some fibers of the septum remaining around the pedicle which were compressing the perforator or the pedicle might not be completely mobilized to the donor vessel. In such cases, further dissection was done to ensure the pedicle vessels were completely mobilized. The perforator dissection to their origin from the donor vessel was not done in all cases. It was only done in cases where there was inadequate mobility and torsion on the perforator due to its short length (Figures 1-5).

In one of our cases of post burn knee contracture, the flap was islanded on two perforators which were very close to each other, rotated through around 90 degrees and we were able to give inset to the flap without tension and kink on the pedicle. The flap was tunneled below the semitendinosus muscle to improve the reach of flap and to facilitate suturing without undue tension. Then tendon lengthening and repositioning of the muscle underneath the flap was done. The flap was monitored for any vascular insufficiency and when none was encountered, it was decided to retain both the perforators (Figure 6).

**Fi.g 6:**
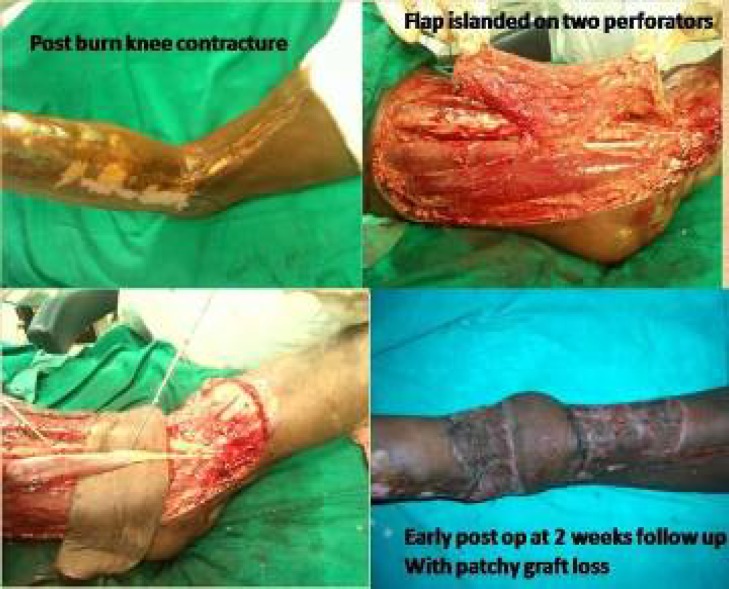
Post burn knee contracture managed by perforator flap

**Fig. 7:**
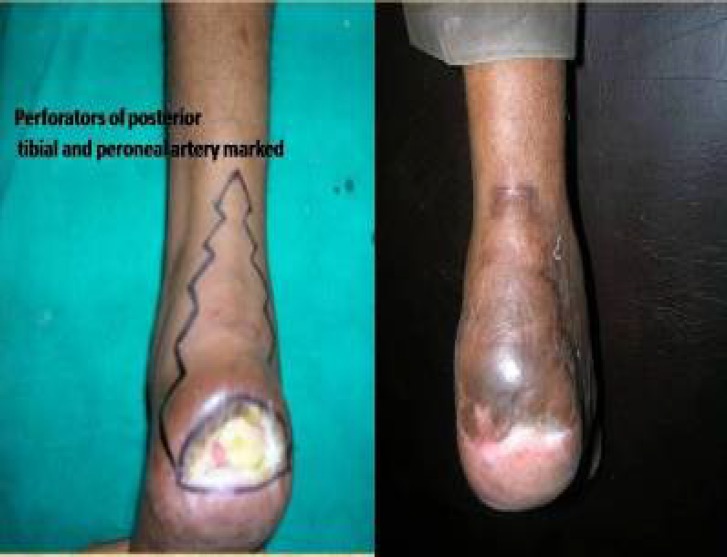
Calcaneal defect managed by perforator flap.

In one case where VY advancement flap was done to cover the calcaneal defect, we retained two perforators– one from the posterior tibial and one from the peroneal artery. This was possible because the two perforators were nearby and could be mobilized to cover the defect. In the post operative period, an anterior slab was given with foot in a minimally dorsiflexed position to prevent tension over the suture line (Figure 7). The Ulnar digital artery perforator flap^3^ was done in three cases of little finger contracture.

The flap was sutured in its new position and the donor area was covered by split skin graft. While suturing the flap, due care was taken that flexor aspect of the joint was covered by the flap. The initial two sutures were placed on the side of the perforators so as to prevent torsion and stretch on the perforator. Stretching and drying are the most common causes of perforator thrombosis during the operation and must be prevented. Twisting of the pedicle occurred more commonly in perforator flaps than in conventional flaps. Paying special attention to the course of the vessels during the inset, were vital maneuvers. Light dressings coverred the flap, which could be monitored easily for any change in color. 

**Fig. 8:**
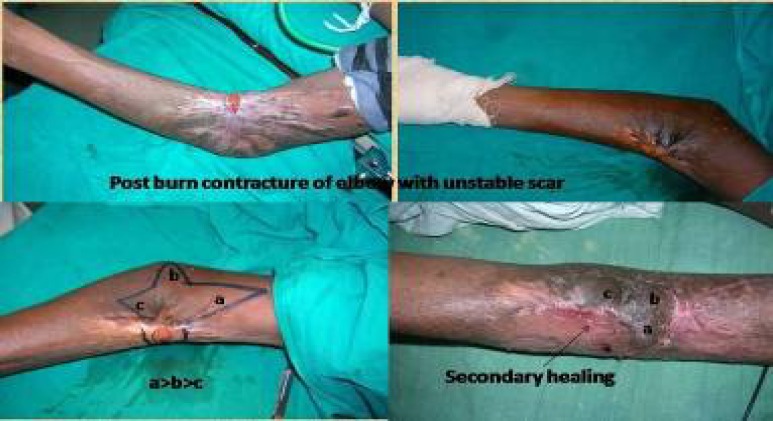
Elbow contracture managed by trilobed perforator flap

**Fig. 9:**
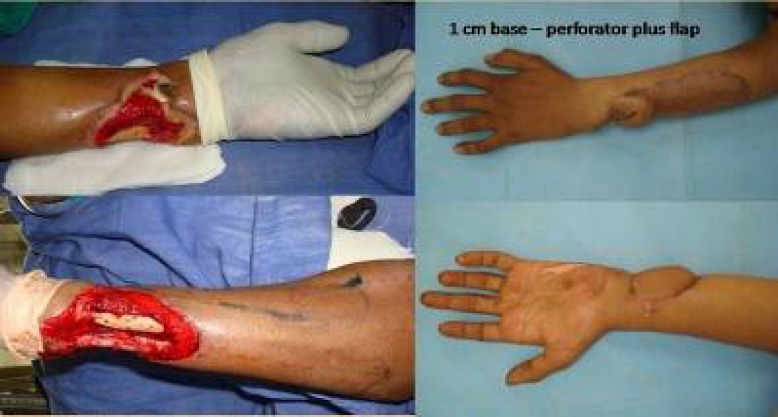
Distal wrist defect managed by perforator plus flap.

**Fig. 10:**
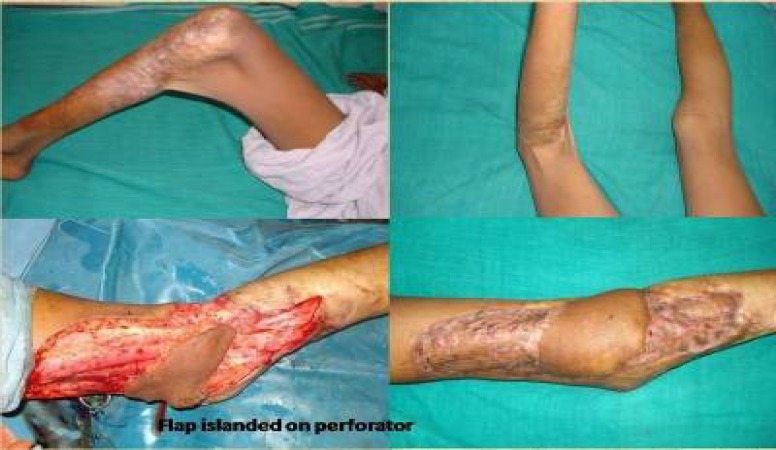
Knee contracture defect covered by genicular artery perforator flap.

A splint was applied which helped immobilization for ten to twelve days postoperatively. Prophylactic antibiotics were given for 3-5 days and the sutures were removed on the 10^th^ day. Pressure dressings in the form of crepe bandage were started from post-operative day seven. Few other clinical cases were shown (Figures 8-10).

Flap was monitored by clinical examination of color, and Doppler study over the perforator. 

The perforator flaps were much more difficult to salvage than conventional free flaps. Once the thrombi were formed, and they migrated to the smaller pedicles, it became almost impossible to salvage the flap. Small amounts of congestion was generally present in cases of perforator flaps; however it tended to subside over a period of time. Strict limb elevation was given in the post-operative period. If on clinical examination there was increased congestion, or the Doppler flow indicated block or decreased flow, it indicated that there was kink on the perforator. Removing the less important sutures relieved the tension on the flap. Compressions of the perforator by an underlying hard surface and twisting or kinking of the perforator may cause cessation of circulation of the flap. Urgent repositioning of the flap can be done to its original position, and reposited back to the defect after the flap settles down.

Important technical considerations in our study were (i) Preoperative marking of the dominant perforator, (ii) Wide exposure of the surgical field and bloodless dissection that were the keys, (iii) Skin island that must be centered on the top of the perforator or following the direction of the main branch of the perforators within the flap, (iv) Dissecting the perforators under loupe or microscopic magnification, (v) Avoiding drying and spasm by constant irrigation with lignocaine and saline solution, (vi) Thin rim of fat that could be left around the perforator for additional support and to prevent kinking, (vii) All fibrous strands that have to be dissected, and the perforator denuded to prevent kinking due to any strand, (viii) The flap that must be sutured in place when the donor site was prepared for grafting, taking care that the entire flexor aspect of the joint was covered by the flap, (ix) The initial sutures that were taken when suturing the perforator flap in place were the two sutures on both sides of the perforator so that there was no kink and torsion on the pedicle, (x) Before transection of the extra perforators, the most dominant perforator was identified by clamping the perforators, (xi) Flap was inset without tension to avoid circulatory disturbances at the distal part and (xii) Hemostasis that must be perfect. If in doubt, drains must be inserted at appropriate places below the flap.

## RESULTS

A total of sixteen patients of post-burn sequelae were operated from November 2008 to April 2010. Follow up ranged from 2 weeks to fifteen months. All patients had burn sequelae like post-burn contractures, post-burn raw areas, post-electric burn raw areas, and marjolins ulcers. A variety of perforator flaps like propeller, perforator based VY advancement and perforator plus flaps were used to cover the defects. All the flaps did well except marginal necrosis in one patient which healed secondarily. As in all perforator flaps, there was minimal amount of congestion of the flap in the early post-operative period, which settled down subsequently. However, in all patients, there was persistent edema of the flap even in the late post-operative period. There was no need of prolonged post-operative splintage and there was no recurrence of the contracture in any of the patients. There was no need of repeat surgery in any of the patients. All patients had significant functional improvement. Most of patients who sustained electrical burns were male and most with flame burns were females. In our series we had maximum number of post burn knee contractures (five) wherein genicular artery based perforator flaps were done to cover the defect. The details of the cases were described in Table 1.

## DISCUSSION

Burn is an alarming problem in the developing world. There are almost 60 to 70 lakh burn cases annually in India alone.^1^ The cost of burn care is enormous, and tertiary care burn centers are ill equipped.^1^ Most of the burn cases in the acute setting are managed at smaller centers by inadequately trained staff resulting in post-burn contractures and deformities.^3^ For optimal burn rehabilitation of the patient, contracture release and coverage of raw areas is mandatory. Whenever contracture release and split skin grafting is done, there is need of prolonged post-operative splintage and physiotherapy for a minimum of six months.^4^^-^^6^

In the developing countries, where most of the burn victims are from the poor socioeconomic strata, postoperative splintage, pressure garments, and change of pressure garment and physiotherapy means loss of money and work hours. The patients are therefore reluctant to use splints in the post-operative period. Thermoplastic and other durable splints which are user friendly are costly are detrimental factors against the use of splints.^7^ The end result being that the patients do no comply to the usage of splints, pressure garments and physiotherapy, and there is a recontracture. Recontracture means the repeat of surgery if it is not manageable by other means like physiotherapy. It means an increasing burden on the government machinery and already collapsing infrastructure.

It is therefore necessary that in this scenario, we need to manage the patients in such a way that there is minimal or no need of post-operative compliance on part of the patient in the form of splintage, physiotherapy, etc. That can be possible by giving a full thickness cover in the form of flaps. Flaps can be local, distant pedicle or free flaps. Generally it is considered that the local tissue is scarred and not suitable to be elevated as a flap for reconstructing the local area.^8^^,^^9^ Therefore, distant flaps are used like groin or abdominal flaps. Groin or abdominal flaps lead to a very cumbersome position to the patient.^10^ These flaps need repeated surgeries, one for attachment of flap and others for detachment of flaps and then for secondary thinning of flaps if the flaps are very bulky.^10^ Free flaps are technically demanding and the facilities and expertise are not universally available.^11^ The use of perforator and propeller flaps for burn reconstruction is relatively sparse in literature. These flaps are raised from the local burn area near the affected site. A perforator is included in the flap which adds to the vascularity of the flap. Thus a flap can be safely raised from the burnt area.^12^^,^^13^ Wherever we have used burnt skin for harvesting a perforator flap, we have always included the deep fascia in addition to the inclusion of the perforator. Isolating the flap on a single perforator gives it an increased arc of mobility, thus reducing the dog ears. It is a safe, reliable, technically much simpler than free flaps, easy to learn, do not have any specific infrastructural needs and can be replicated with relative easy. If the perforator flap is used in fashion of a propeller flap, part of the flap covers the donor area and the need for skin graft is also reduced and needless to say donor site is restricted to a single body area. As we provided a full thickness cover, there is no risk of recurrence of contracture, there is no need of post-operative splintage and patient may return back to work soon. The need of graft is also reduced, and the graft is not applied over the flexural aspect of the joints thereby, reducing the risk of contractures.

Perforator propeller flaps are an important addition to the armamentarium of the plastic surgeon for the management of burn wound and sequelae. These flaps are simple, safe, and versatile and must be considered as the primary option for treatment of burn wounds and sequel wherever possible.

## CONFLICT OF INTEREST

The authors declare no conflict of interest.
